# Ribonucleotide Reductase Subunit M2 Predicts Survival in Subgroups of Patients with Non-Small Cell Lung Carcinoma: Effects of Gender and Smoking Status

**DOI:** 10.1371/journal.pone.0127600

**Published:** 2015-05-22

**Authors:** Vei Mah, Mohammad Alavi, Diana C. Márquez-Garbán, Erin L. Maresh, Sara R. Kim, Steve Horvath, Lora Bagryanova, Sara Huerta-Yepez, David Chia, Richard Pietras, Lee Goodglick

**Affiliations:** 1 Department of Pathology and Laboratory Medicine, UCLA, Los Angeles, California, 90095, United States of America; 2 Department of Medicine, Division of Hematology-Oncology, UCLA, Los Angeles, California, 90095, United States of America; 3 Department of Biostatistics, UCLA, Los Angeles, California, 90095, United States of America; 4 Department of Human Genetics, UCLA, Los Angeles, California, 90095, United States of America; 5 Jonsson Comprehensive Cancer Center, David Geffen School of Medicine at UCLA, Los Angeles, California, 90095, United States of America; 6 Unidad de Investigación en Enfermedades Oncológicas, Hospital Infantil de México, Federico Gómez, SSa, México; H. Lee Moffitt Cancer Center & Research Institute, UNITED STATES

## Abstract

**Background:**

Ribonucleotide reductase catalyzes the conversion of ribonucleotide diphosphates to deoxyribonucleotide diphosphates. The functional enzyme consists of two subunits - one large (RRM1) and one small (RRM2 or RRM2b) subunit. Expression levels of each subunit have been implicated in prognostic outcomes in several different types of cancers.

**Experimental Design:**

Immunohistochemistry for RRM1 and RRM2 was performed on a lung cancer tissue microarray (TMA) and analyzed. 326 patients from the microarray were included in this study.

**Results:**

In non-small cell lung cancer (NSCLC), RRM2 expression was strongly predictive of disease-specific survival in women, non-smokers and former smokers who had quit at least 10 years prior to being diagnosed with lung cancer. Higher expression was associated with worse survival. This was not the case for men, current smokers and those who had stopped smoking for shorter periods of time. RRM1 was not predictive of survival outcomes in any subset of the patient group.

**Conclusion:**

RRM2, but not RRM1, is a useful predictor of survival outcome in certain subsets of NSCLC patients.

## Introduction

Lung cancer continues to be the major cause of cancer mortality in the United States, both in men and women [1]. Although the majority of cases of non-small cell lung cancer (NSCLC) are in smokers and former smokers, approximately 10%- 40% occur in non smokers. The percentage varies by geographic region, with higher percentages occurring in non smokers in Asia. Differences in genetic patterns and outcomes have been noted in NSCLCs derived from non-smokers compared to smokers [2, 3] as well as men compared to women [4]. Govindan et al. found a more than 10-fold higher average mutation frequency in smokers than non smokers. Mutations more often encountered in non smokers such as EGFR mutations, and ROS1 and ALK fusions differed from smokers who showed higher rates of KRAS, TP53, BRAF, JAK2, JAK3 and mismatch repair gene mutations [2, 5].

The enzyme ribonucleotide reductase (RNR) catalyzes the conversion of ribonucleotide diphosphates to deoxyribonucleotide diphosphates prior to DNA synthesis in dividing cells. One large 90 kD subunit (RRM1) and two small subunits (RRM2 and RRM2b/p53R2) have been identified in humans. The active enzyme is an oligomer of large and small subunits in the configuration α_n_β_n_. Minimally n must be two for a functional enzyme [6, 7]. The RRM1 subunit contains two allosteric sites along with a catalytic domain. The catalytic domain on RRM1 is formed only in the presence of the RRM2 subunit [7, 8]. The small subunit contains sites for binding of two irons and a tyrosyl radical necessary for enzyme activity [9]. RRM1 levels are in excess of RRM2 and relatively constant throughout the cell cycle [8, 10]. Levels of RRM2 are cell cycle dependent, with highest levels during S-phase [11, 12], while RRM2b expression is upregulated by various genotoxic events. RRM2b is p53 inducible and plays a pivotal role in repair of DNA damage [13]. It is also necessary for mitochondrial DNA maintenance [14]. RNR is important for regulating sizes of dNTP pools, which in turn is important for correct DNA replication [14]. Changes in the size of dNTP pools or their balance can lead to increased mutation rates [14, 15]. Xu et al. found that overexpressing RRM2 in transgenic mice induced lung neoplasms with K-ras being a frequent mutational target [16].

Expression levels of the different ribonucleotide reductase subunits have been studied in various cancers. Aye et al. found RRM2 was among the top 10% of most overexpressed genes in 73/168 cancers and RRM1 was among the top 10% in 30/170 cancers [9]. Possibly elevated RNR subunit expression may be a reflection of increased numbers of cancer cells in S phase. In early stage non small cell lung cancer, Hsu et al. [17, 18] found RRM2 correlated positively with tumor grade and patients with RRM2- and RRM2b+ tumors had better outcomes. In their study RRM2b was a better predictor for both recurrence and survival than RRM2. In colorectal cancer, Lu et al. [19] found RRM2 levels correlated with invasion depth, poorer differentiation, and tumor metastasis and Liu et al. [20] found higher RRM2 also to be associated with metastases as well as worse survival. In gastric cancer, Morikawa et al. [21] found RRM2 overexpression (>10%) in 64% of tumors and this correlated with *muscularis propria* invasion, male gender and survivin expression, but not with age, histology, tumor size or lymph node metastasis. Higher levels of RRM2b were associated with improved survival in colorectal cancer [22] as well as early stage NSCLC. However, in melanoma patients [23], RRM2b correlated positively with depth of invasion and tumor stage.

Ribonucleotide reductase inhibitors have been studied and used as chemotherapeutic agents and as radiation sensitizers [24]. Ribonucleotide reductase inhibitors used in cancer therapy include hydroxyurea, fludarabine, cladribine, gemcitabine, tezacitabine, and triapine. In several studies, RRM1 levels were found to be inversely correlated with tumor response to gemcitabine treatment [25–28], and increased RRM1 expression was associated with gemcitabine resistance in various cell lines [29, 30]. In some studies of patients with NSCLC and other cancers, low RRM1 in patients treated with surgery was associated with reduced survival, but if treatment with gemcitabine was given, low RRM1 was associated with improved survival [30]. Thus endogenous variations in ribonucleotide reductase activity in tumor cells may be useful in determining response to agents with inhibitor activity for this enzyme.

Previously, in a meta-analysis of eight DNA microarray datasets, we identified RRM2 as part of a 63 gene signature strongly related to survival outcome in lung adenocarcinomas [31]. Because of those results, we decided to examine in more detail the expression of RRM2 on a NSCLC tissue microarray by immunohistochemistry. In addition, we studied RRM1 expression because the RRM1 subunit directly interacts with RRM2 and is needed for a fully functional enzyme. We found high RRM2 levels predicted overall disease specific survival in certain subsets of the patient population more strongly than others. In women, non-smokers and former smokers who had quit smoking for at least 10 years, higher cytoplasmic RRM2 levels correlated with significantly worse survival. In contrast, RRM1 was neither predictive of survival in the entire patient population nor any patient subgroup. Combined expression of RRM1 and RRM2 did not predict patient outcomes better than RRM2 alone.

## Materials and Methods

### Patient material

The lung cancer tissue microarray (TMA) was constructed with archival formalin-fixed, paraffin embedded lung cancer tissue samples from the UCLA Department of Pathology and Laboratory Medicine under appropriate Institutional Review Board and Health Insurance Portability and Accountability Act regulations. The array has been described previously [32, 33]. Briefly, the entire array contained tissue from 696 surgical specimens from 671 patients. Tissues sampled included primary lung tumor, adjacent normal appearing lung parenchyma and metastatic lung carcinoma to lymph nodes and distant sites. All cases had been reviewed by at least two pathologists for confirmation. At least three 0.6 mm core tissue biopsies from each case were taken from the represented morphologies as previously described.

For analyses, 326 of the 671 patients had adequate tissue information for both RRM1 and RRM2 expression in the primary lung neoplasm as well as clinical information and survival outcomes. Metastatic and recurrent tumors and small cell carcinomas were excluded from this particular analysis. Patients with any history of neoadjuvant therapy were also excluded. Of the 326 patients, 169 were women, and 157 were men. 201 cases were adenocarcinoma (113 women, 88 men) and 83 were squamous cell carcinoma (34 women, 49 men). The breakdown of other histologies is listed in Table 1. Median age of patients was 67 years for men and 68 years for women. Non-smokers were those who had less than 100 cigarettes over their lifetime. Former smokers were those who had quit at least one year prior to surgery. A more detailed listing of demographic differences is presented in Table 1.

**Table 1:** Differences in expression levels of cytoplasmic RRM1 and RRM2 (integrated mean intensity) by different subgroups.

group	n	RRM1 (mean ± SEM)	p-value	RRM2 (mean ± SEM)	p-value
gender			0.655§		0.861§
women	169	0.536 ± 0.038		0.147 ± 0.016	
men	157	0.571 ± 0.042		0.151 ± 0.018	
stage			0.501‡		0.496‡
stage I	193	0.530 ± 0.035		0.144 ± 0.016	
stage II	57	0.503 ± 0.061		0.126 ± 0.020	
stage III	52	0.615 ± 0.072		0.194 ± 0.035	
stage IV	22	0.724 ± 0.155		0.156 ± 0.050	
grade			0.266‡		< 0.0001
grade 1	52	0.450 ± 0.065		0.040 ± 0.012	
grade 2	91	0.641 ± 0.058		0.128 ± 0.024	
grade 3	132	0.576 ± 0.041		0.200 ± 0.019	
grade 4	22	0.585 ± 0.146		0.265 ± 0.065	
histology			0.001‡		< 0.0001
adenocarcinoma	201	0.472 ± 0.032		0.100 ± 0.012	
adenosquamous carcinoma	14	0.658 ± 0.139		0.188 ± 0.064	
squamous cell carcinoma	83	0.740 ± 0.060		0.240 ± 0.028	
large cell carcinoma	20	0.580 ± 0.157		0.274 ± 0.071	
smoking status			0.851‡		0.013‡
non-smokers	44	0.572 ± 0.085		0.109 ± 0.030	
former smokers	132	0.532 ± 0.041		0.132 ± 0.017	
current smokers	90	0.588 ± 0.057		0.183 ± 0.026	
ethnicity			0.5‡		0.118‡
African-American	11	0.628 ± 0.237		0.115 ± 0.048	
Asian American	28	0.705 ± 0.113		0.151 ± 0.046	
Caucasian	241	0.527 ± 0.030		0.160 ± 0.014	
Hispanic	6	0.549 ± 0.253		0.026 ± 0.023	

^‡^ Kruskal-Wallis test.

^§^ Mann-Whitney U test.

### Immunohistochemistry

The lung TMA was stained using a standard two-step indirect immunohistochemical procedure as previously described. Initial steps before application of primary and secondary antibodies have been described previously [33]. The primary antibody for detecting RRM1 was a rabbit anti-RRM1 polyclonal antibody (product #ab81085 from Abcam, Cambridge, MA; http://www.abcam.com/rrm1-antibody-ab81085.html) against the C-terminus of human RRM1. This antibody was applied overnight at 4°C at 1:100 dilution. For RRM2, primary goat anti-RRM2 antibody (E-16) (product sc-10846 from Santa Cruz Biotechnology, Dallas, TX; http://www.scbt.com/datasheet-10846-r2-e-16-antibody.html) was applied for 45 minutes at room temperature at 1:400 dilution. For detection the Dako Envision System was used, with diaminobenzidine (DAB) for chromogen visualization. Slides were counterstained with Harris’ haematoxylin. Negative controls were identical sections stained without application of the primary antibody. Semiquantitative assessment of RRM1 and RRM2 staining on the TMA was performed by a pathologist (M. Alavi) without concurrent knowledge of clinical information. A number value was calculated based on staining intensity of individual cells (0 = not detectable, 1 = weak; 2 = moderate; and 3 = strong) and percentage of cells staining at each intensity level (0–100%). A final integrated value of intensity and frequency was derived by the following formula: [(3x) + (2y) + (1z)] / 100 where x, y, and z are % staining at intensity 3, 2, and 1, respectively. This value was then used to compare tissue staining among patients [33].

### Statistical analysis

The open source R software (http://www.R-project.org) including survival and Hmisc packages was used for data analysis. Pooling criteria have been described before [33]. For the current studies we used a mean average over all the appropriate cores for a given patient. Differences in expression among the different subgroups were tested with the Wilcoxon signed rank test or Kruskal-Wallis rank sum test. Survival curves were calculated using the Kaplan-Meier method and comparisons made with the log-rank test. The Cox proportional hazards model (univariate and multivariate) was used to determine the significance of various factors related to survival. P-values were two-sided and those < 0.05 were considered significant. Data tables used for analyses are submitted as S1, S2 and S3 Tables.

### Ethics Statement

IRB approval was obtained through the UCLA Office of Human Research Protection Program (IRB#11-00-1301). All patient data was de-identified before experiments were performed. IRB waived the need for informed consent.

## Results

### Expression pattern of RRM1 and RRM2 in NSCLC

**Fig 1:**
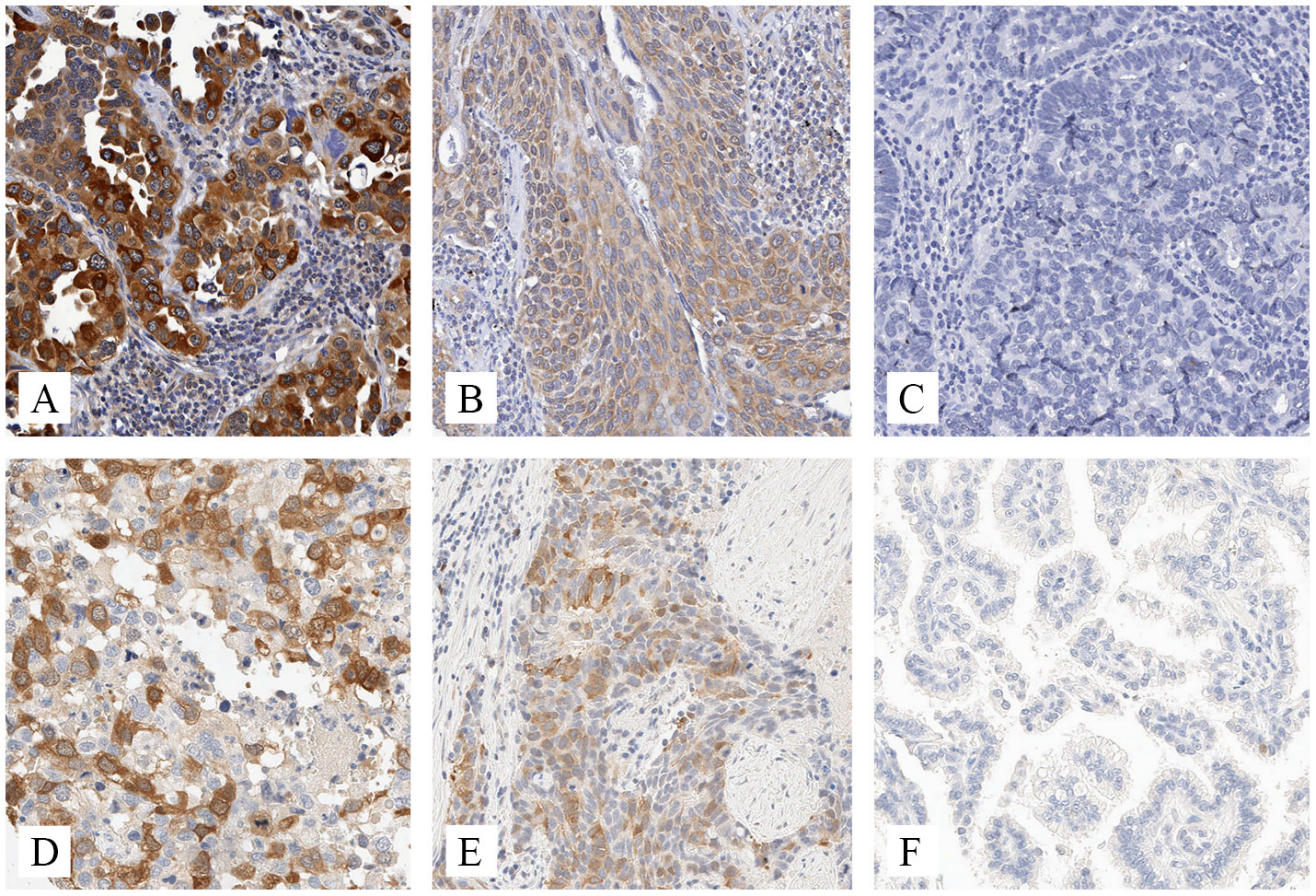
Photomicrographs of RRM1 and RRM2. Representative images (20x magnification) showing cellular variability for immunohistochemical staining of (A-C) RRM1 and (D-F) RRM2. Many tumors showed completely negative staining (C and F), while others showed variable staining of individual cells within the tumor (A,D,E) and some showed more uniform staining of individual tumor cells (B).

**Fig 2:**
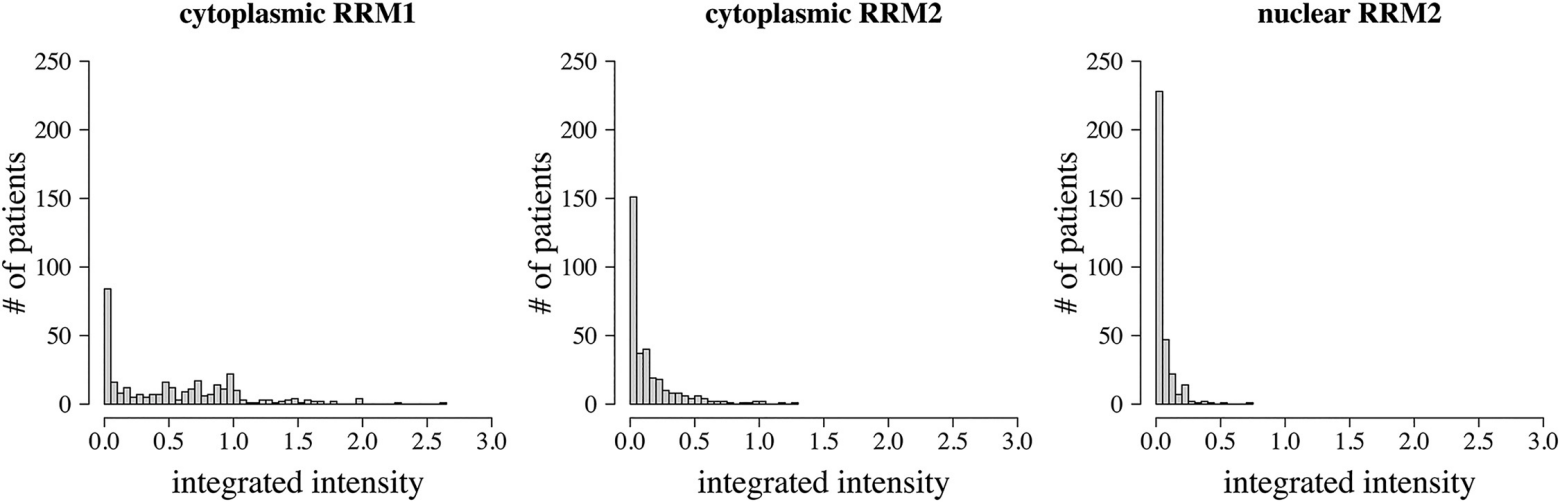
Histograms of RRM1 and RRM2 staining patterns. Distribution for staining intensity (measured by integrated intensity: [(3x) + (2y) + (1z)] / 100 where x, y, and z are % staining at intensity 3, 2, and 1, respectively) of (A) cytoplasmic RRM1, (B) cytoplasmic RRM2 and (C) nuclear RRM2. Although variable, overall staining was stronger for cytoplasmic RRM1. Many patients showed weak or negative staining.

Although many tumors were negative or weakly stained for both RRM1 and RRM2, RRM1 staining tended to be slightly stronger than RRM2. RRM1 showed predominantly cytoplasmic immunoreactivity. RRM2 immunoreactivity was seen in both nuclear and cytoplasmic compartments; however cytoplasmic RRM2 staining tended to be more intense. Nuclear and cytoplasmic levels of RRM2 were highly correlated (Spearman rho = 0.72, p < 10^–5^). There was weak correlation between RRM1 and RRM2 staining intensities (rho = 0.265, p < 10^–5^). Representative photographs showing the variability of RRM1 and RRM2 staining are presented in Fig 1. Overall distribution of staining intensities across all tumors is shown in Fig 2.

### Correlation of RRM1 and RRM2 expression with clinical parameters

**Fig 3:**
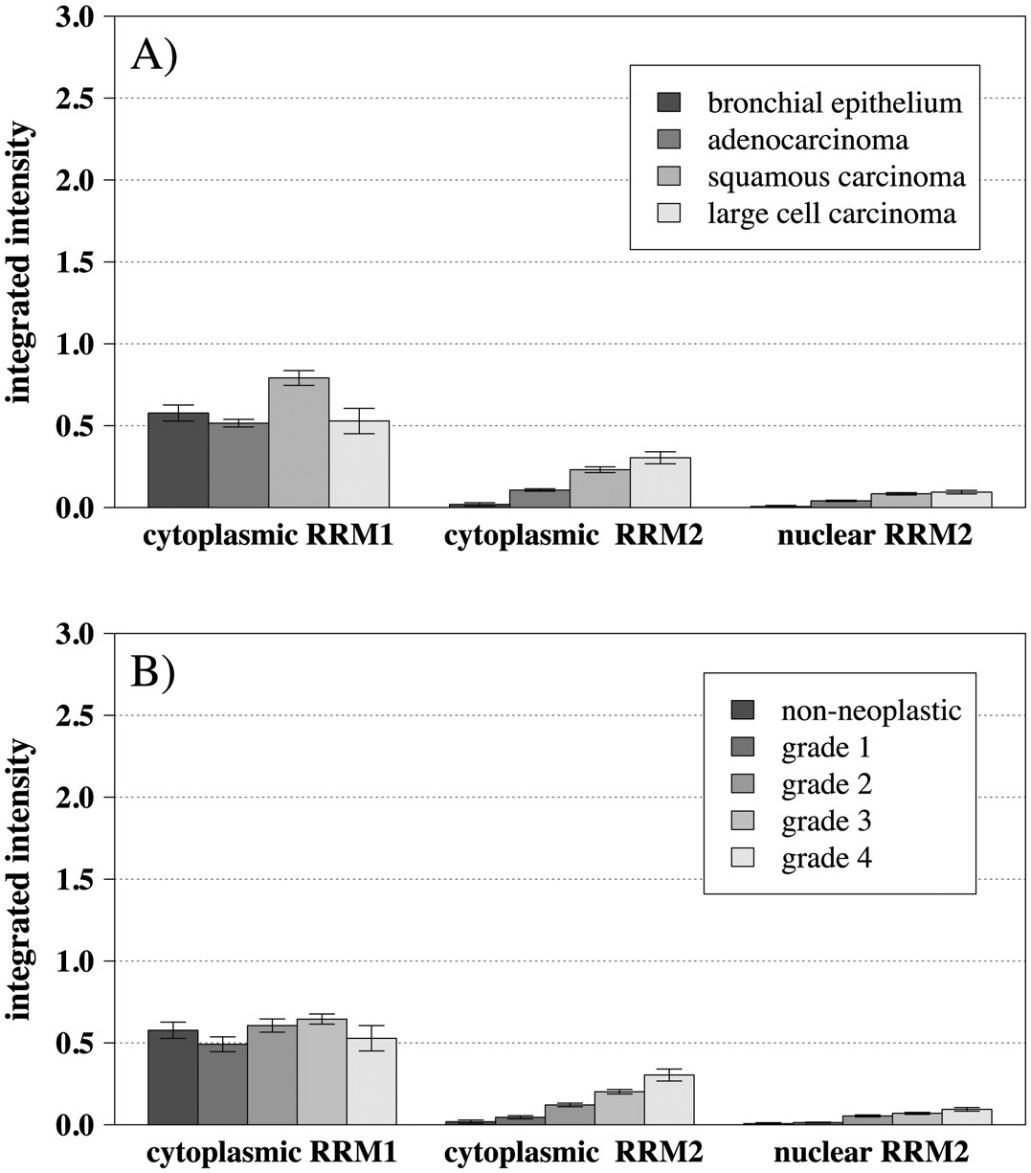
Barplots for relative RRM1 and RRM2 protein expression levels in cytoplasm (integrated intensity, scale from 0 to 3) by (A) histology and (B) grade. Both RRM1 and RRM2 expression were higher in squamous carcinoma than adenocarcinoma (p < 10^–5^). RRM1 expression did not correlate with tumor grade (rho = 0.04, p = 0.55) while RRM2 expression correlated positively with grade (rho = 0.44, p < 10^–5^ for cytoplasm and rho = 0.36, p < 10^–5^ for nucleus).

Parameters examined included stage, presence of metastases, grade, histology, smoking status and age. RRM2 expression correlated positively with tumor grade (rho = 0.44, p < 10^–5^ for cytoplasm and rho = 0.36, p < 10^–5^ for nucleus). For expression levels by histology, we found that squamous cell carcinomas tended to have higher levels of both RRM1 (p < 10^–5^) and RRM2 (p < 10^–5^ cytoplasmic and nuclear) than adenocarcinomas (Fig 3A). There was significantly increased expression in cancer compared to normal appearing adjacent bronchial epithelium for both nuclear and cytoplasmic RRM2 (p < 10^–5^, Fig 3A). This was not seen for RRM1 (p = 0.79). Higher grade was also associated with higher expression of RRM2 (rho = 0.44, p < 10^–5^ for cytoplasm and rho = 0.36, p < 10^–5^ for nucleus) but not RRM1 (rho = 0.04, p = 0.55, Fig 3B). Both RRM1 and RRM2 also correlated weakly with number of smoking pack years (p = 0.006, rho = 0.171 for RRM1; p = 0.001, rho = 0.204 for cytoplasmic RRM2 and p = 0.0086, rho = 0.164 for nuclear RRM2). Otherwise, there was no correlation with smoking status (current, former and non smokers), tumor stage or presence of metastases. Details are listed in Table 1.

### Correlation with survival outcomes

**Fig 4:**
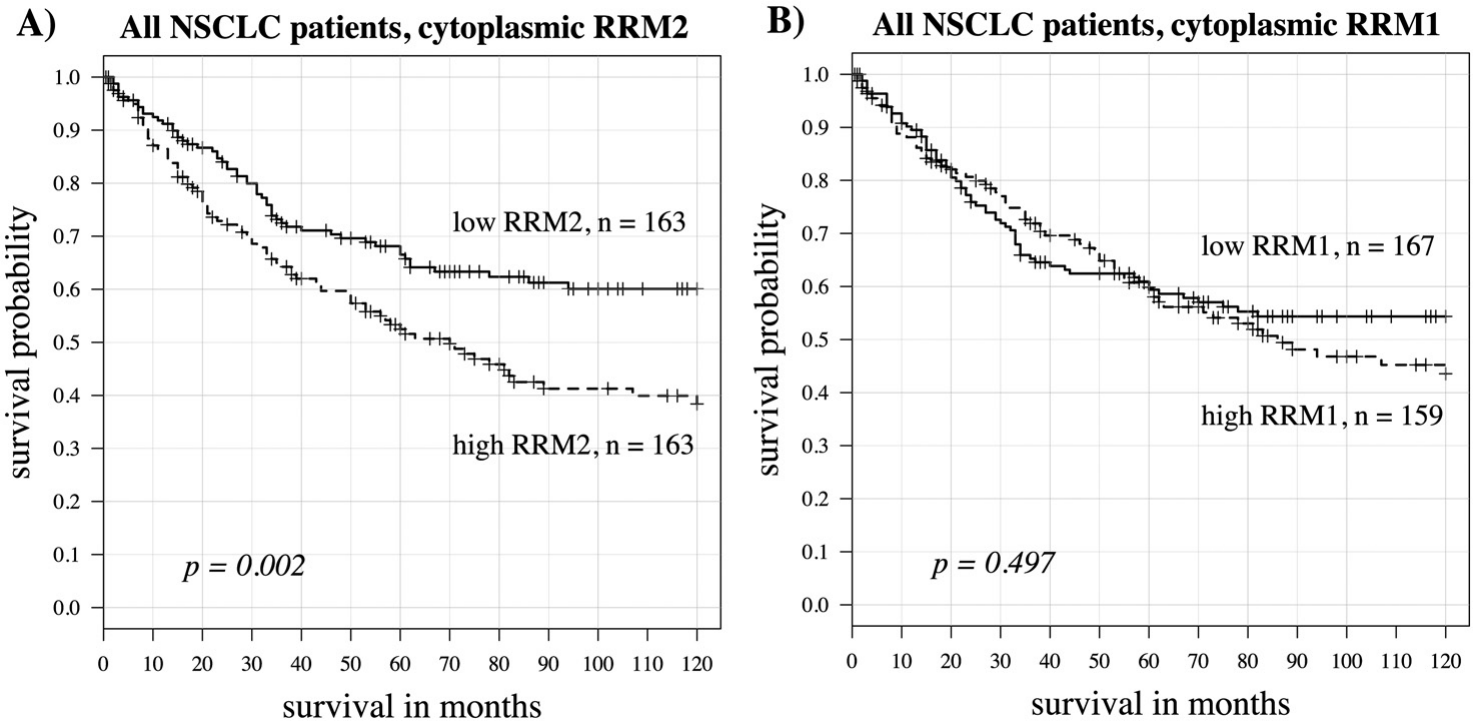
Kaplan Meier curves for RRM1 and RRM2 in all patients. (A) cytoplasmic RRM2, split by median expression levels (p = 0.002, hazard ratio = 1.70). (B) cytoplasmic RRM1, split by median expression levels (p = 0.497, hazard ratio = 1.12).

**Fig 5:**
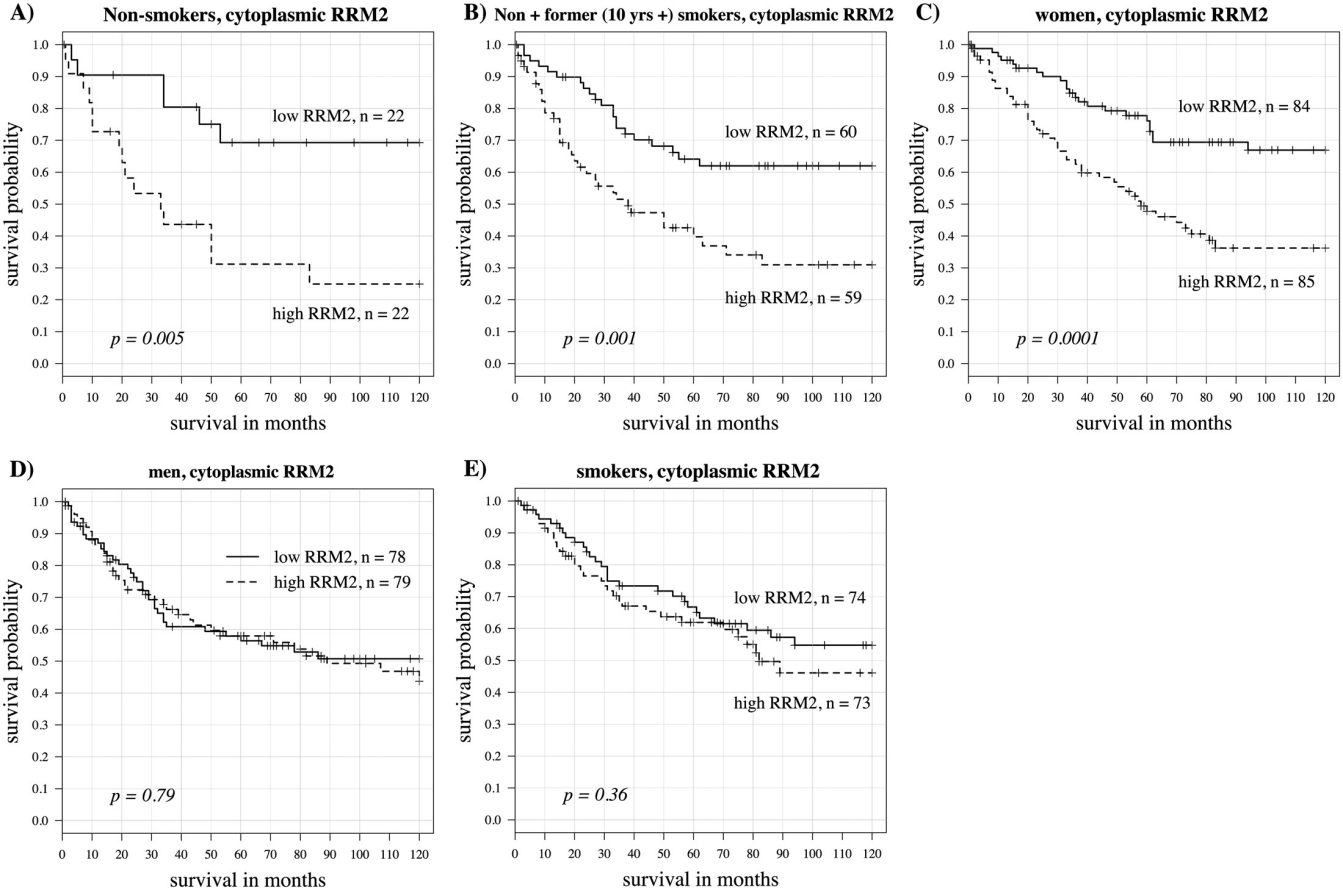
Kaplan Meier curves for cytoplasmic RRM2 split by median expression levels in patient subgroups. (A) non-smokers (p = 0.005, hazard ratio = 3.58). (B) non-smokers plus those quitting more than 10 years (p = 0.001, hazard ratio = 2.38). (C) women (p = 0.0001, hazard ratio = 2.57). (D) men (p = 0.79, hazard ratio = 1.07). (E) smokers—includes current smokers plus those quitting 10 years or less (p = 0.36, hazard ratio = 1.27).

**Fig 6:**
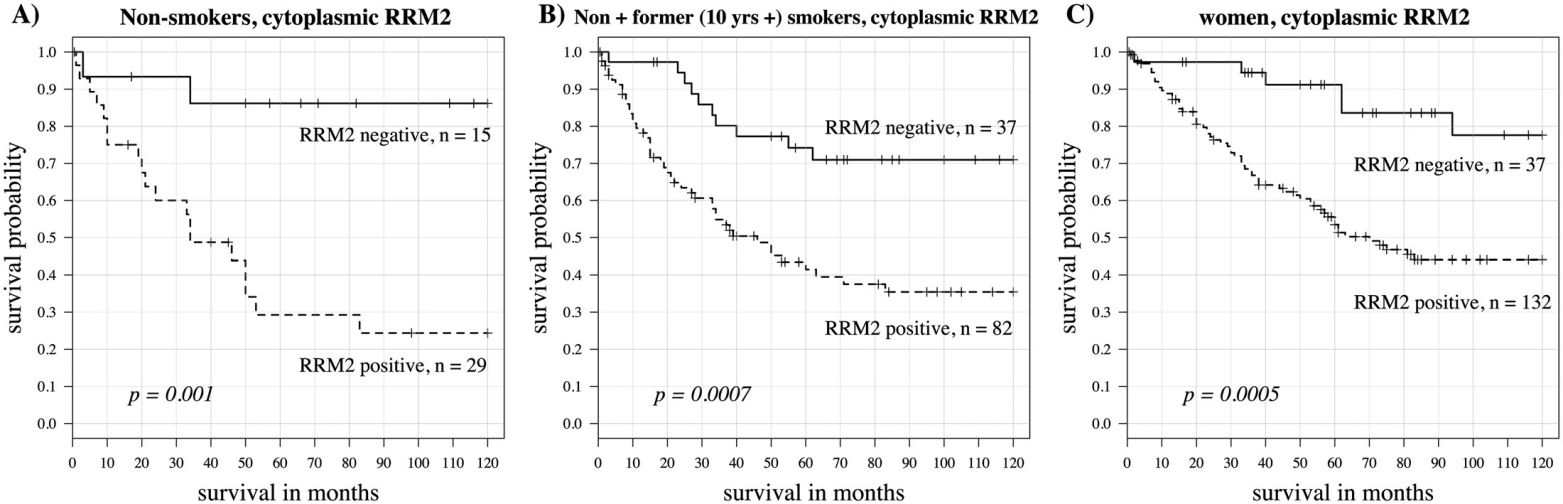
Kaplan Meier curve for cytoplasmic RRM2 split by positive vs. negative staining. (A) non-smokers (p = 0.001, hazard ratio = 7.81). (B) non-smokers plus those quitting more than 10 years (p = 0.0007, hazard ratio = 3.11). (C) women (p = 0.0005, hazard ratio = 3.99).

In the whole population of 326 patients, cytoplasmic RRM2 was a significant predictor of survival, with higher levels predicting worse outcomes (univariate Cox model log rank p = 0.002 and hazard ratio = 2.73) A Kaplan-Meier curve using the median value as the cut point is shown in Fig 4A. Nuclear RRM2 expression, although highly correlated with cytoplasmic expression, was not a significant survival predictor here. Levels of RRM1 expression did not predict survival outcome. A representative Kaplan-Meier curve is shown in Fig 4B. The patient population was then subdivided by gender, smoking status, age, stage and histology. Here we found gender and smoking status to be important variables in determining the utility of cytoplasmic RRM2 as a predictor for survival. In current smokers (including those quitting smoking less than one month prior to diagnosis), RRM2 was not predictive for survival (p = 0.85, hazard ratio = 1.13). However, in non-smokers, high RRM2 expression was associated with worse survival (p < 10^–5^, hazard ratio = 54.29). In former smokers, high RRM2 expression was also associated with poor survival (p = 0.002, hazard ratio = 5.63). On further subdividing former smokers, RRM2 was a significant predictive variable for survival only in those patients quitting over 10 years prior to diagnosis (p = 0.001, hazard ratio = 12.75). In those quitting less than 10 years, p = 0.25 and hazard ratio = 2.56 by the univariate Cox model. In women (both older and younger age groups), RRM2 also significantly predicted worse survival (p = 0.0002 and hazard ratio = 5.11) but this was not the case in men (p = 0.413 and hazard ratio = 1.51). When examining survival related to nuclear expression of RRM2, findings were similar except not as strong (Table 2). RRM1 was not a significant survival predictor in any of these patient subgroups. Kaplan-Meier curves for cytoplasmic RRM2 in these subgroups using the median value cut point are shown in Fig 5. Kaplan-Meier curves showing positive (staining seen in any cell) vs negative (no detectable staining) are shown in Fig 6. Table 2 shows predictive value (p-values and hazard ratios) for cytoplasmic RRM1, cytoplasmic RRM2 and nuclear RRM2 as continuous variables in a univariate Cox model. Cytoplasmic RRM2 remained an independent predictor of survival in the multivariate Cox model, taking into account stage, age and grade (Tables 3 and 4).

**Table 2:** Univariate Cox model for cytoplasmic RRM1, cytoplasmic RRM2 and nuclear RRM2 as predictors of survival in patient subgroups.

patient group	n	RRM1 p	RRM1 HR	cytoplasmic RRM2 p	cytoplasmic RRM2 HR	nuclear RRM2 p	nuclear RRM2 HR
all patients	326	0.238	1.22	2.05E-03	2.73	0.145	3.17
women	169	0.220	1.36	1.95E-04	5.11	1.86E-03	51.81
men	157	0.657	1.10	0.413	1.51	0.855	0.81
current smokers	90	0.983	1.01	0.708	1.26	0.847	1.31
former smokers, quit ≤ 10 yrs	57	0.861	1.09	0.329	2.60	0.788	3.09
former smokers, quit > 10 yrs	75	0.122	1.59	1.05E-03	12.75	0.045	99.71
non-smokers	44	0.659	1.22	2.00E-06	54.29	0.020	31.30
current + quitting ≤ 10 yrs	147	0.909	1.03	0.392	1.55	0.688	1.67
non-smokers + quit >10 yrs	119	0.130	1.46	0	21.37	2.65E-03	35.65

**Table 3:** Multivariate Cox proportional hazards model for non-smokers and those quitting 10 years or more.

Variable	Hazard Ratio (95% confidence interval)	p-value
tumor stage	1.69 (1.29–2.23)	0.0002
tumor grade	1.21 (0.83–1.76)	0.32
age	1.02 (0.98–1.05)	0.31
cytoplasmic RRM2	7.47 (1.99–28.11)	0.003

**Table 4:** Multivariate Cox proportional hazards model for women.

Variable	Hazard Ratio (95% confidence interval)	p-value
tumor stage	1.78 (1.38–2.30)	9.95E-06
tumor grade	1.14 (0.81–1.59)	0.46
age	1.02 (0.995–1.05)	0.11
cytoplasmic RRM2	4.47 (1.67–11.96)	0.003

Combining expression of RRM1 with RRM2 did not predict patient outcomes better than RRM2 alone. S1 Fig illustrates this with representative Kaplan-Meier curves for the different subgroups.

## Discussion

Several studies have previously been performed on RRM1, RRM2 and RRM2b expression and outcomes in NSCLC. However, most studies examined the NSCLC population as a whole, without separating by gender and/or smoking status, for which differences in biological properties have been noted [5, 34]. In this paper, we reported immunohistochemical studies of RRM1 and RRM2 on a NSCLC tissue microarray. The stronger staining in cytoplasmic vs. nuclear compartments is consistent with previous studies of RNR being a constitutively cytosolic enzyme [9, 35]. High RRM2 levels (mainly cytoplasmic) predicted significantly worse overall survival in women, non-smokers and former smokers who had quit smoking at least 10 years prior. RRM2 was not a significant predictor of survival in men, current smokers and former smokers who had quit smoking more recently. In contrast, RRM1 was neither predictive of survival in the entire patient population nor any patient subgroup and the combination of RRM1 and RRM2 expression together did not add to the informative value of RRM2 alone.

There are three classes of RNRs based mostly on their interaction with oxygen and the way they generate their thiyl radical [6]. Eukaryotic RNRs mostly belong to class I. RNR tightly controls the *de novo* synthesis of dNTPs, an important step necessary for DNA replication and repair. Ribonucleotide reductase activity itself is controlled by various mechanisms including transcriptional regulation, post-transcriptional modifications, allosteric binding sites on the RRM1 subunit, interaction with the RRM2 or RRM2b small subunit and cellular localization of the enzyme. Over-accumulation or an inappropriate balance of dNTP levels can lead to increased mutation rates, probably at least in part by indirect inhibition of proofreading mechanisms [8]. The N-terminus of RRM1 contains an ATP-binding cone domain. Binding of ATP at this site activates RNR whereas binding of dATP inhibits activity [36]. Oligomerization of RRM1 is required for ATP/dATP regulation. A second allosteric site monitors the balance of dNTPs and adjusts its activity to maintain their ratios [15]. RRM1 levels remain relatively constant throughout the cell cycle whereas RRM2 is variable with highest levels during S phase. Concurrent with RRM2 levels, dNTP pools also vary during the cell cycle with highest concentrations during S-phase [7, 37].

Since the active enzyme is a hetero-dimeric multimer complex, synthesis and degradation of RRM2 can play a crucial role in enzyme function. Two transcription start sites have been identified for the RRM2 gene resulting in cDNAs of 3.4 and 1.65 kb. These have identical coding regions and differ only in the length of untranslated regions [38]. Variability in transcription initiation may exist between the two promoter sites and may be associated with hydroxyurea or gemcitabine resistance in some cells [39]. E2F binding sites as well as other transcription factor binding motifs have been identified in the promoter regions of the gene [38]. E2F4 binding represses RRM2 transcription during G1 phase [6, 40]. Although transcription of both RRM1 and RRM2 peaks during S phase, RRM1 is more stable with a longer half life. After S phase, RRM2 is degraded through anaphase promoting complex-Cdh1 and Skp1/cullin/F-box ubiquitin ligase complex [9]. D’Angiolella et al. found that following CDK-mediated phosphorylation of Thr33 during G2 phase, RRM2 was degraded via SCF^cyclin F^ and this functioned to maintain balanced dNTP pools and genome stability [10]. During mitosis/G1 phase, RRM2 was found to be a target of anaphase promoting complex-Cdh1 mediated proteolysis in mice [41].

Other effects of RRM2 have been noted. Duxbury & Whang found RRM2 induced NFκB activation of MMP9 and enhanced cellular invasiveness in pancreatic adenocarcinoma cells [42]. Tang et al. found that RRM2 also affected Wnt signaling. RRM2 functioned downstream of β-catenin to inhibit Wnt signaling but phosphorylation on serine 20 of RRM2 countered this effect [43]. Shetty et al. found RRM2 was a binding protein for urokinase-type plasminogen activator (uPA) mRNA 3'UTR in pulmonary epithelial cells [44], so RRM2 could affect a range of functions in lung inflammation and repair related to uPA. Therefore prognostic relevance in cancer survival could also be separate from ribonucleotide reductase activity. Other enzymes and proteins also have roles in modulating levels of dNTPs but not necessarily in their direct synthesis. Some of these include CTP synthetase, thymidylate synthase, dihydrofolate reductase, IMP dehydrogenase and DNA methyltransferase. In particular thymidylate synthase, which catalyzes the methylation of deoxyuridylate to deoxythymidylate, has been shown to be elevated in several different cancers [45].

Poor prognostic implications of high RRM2 expression is of interest since it may indicate which subsets of NSCLC patients could benefit more from antimetabolite or siRNA therapy [46] that interferes with RNR function. RNR inhibitors can be classified as translational, dimerization or catalytic inhibitors, depending on their mechanism of action [45]. Binding of allosteric effectors controls RNR activity. Small-nucleotide inhibitors of RNRs fall into two major categories—nucleoside analogs which target RRM1 and redox active metal chelators which target RRM2. Gemcitabine (Gemzar F_2_C) is a fluorinated deoxycytidine analog. It is one of the major chemotherapeutic agents used to treat NSCLC. It is a prodrug converted by deoxycytidine kinase to 5'-diphosphate (dFdCDP) and triphosphate (dFdCYP). dFdCDP inhibits RNR and dFdCTP competes with dCTP for incorporation into replicating DNA [47]. That NSCLC patients with lower levels of RRM1 had better survival with gemcitabine treatment than those with higher levels of RRM1 in some studies [25–28] is not easily explained mechanistically at this point. In addition, to inhibiting RNR, gemcitabine also induces formation of reactive oxygen species (ROS) [48]. Another nucleoside prodrug in clinical use that targets the RRM1 subunit is clofarabine (CIF) which belongs to a class of nucleoside analogs which includes cytarabine, nelarabine, azacitidine, decitabine, cladribine and fludarabine. Complications with use of nucleoside analogs are problematic. Most are administered as prodrugs requiring phosphorylation for activation. Variable drug metabolism can lead to steady state levels of active forms with variable toxicity and side effects. Inhibitors of RRM2 include hydroxyurea (HU) and triapine. HU is a metal chelator and radical quencher [9]. Although it targets RRM2, overexpression of RRM2 confers resistance in mouse cells. HU is not specific as it also targets other metalloenzymes such as carbonic anhydrase and matrix metalloproteinases. In addition to different cancers, it is also used in the treatment of sickle cell anemia, polycythemia vera, AIDS and other conditions. Triapine (3-aminopyridine-2-carboxaldehyde thiosemicarbazone) can synergize with other antitumor drugs that target DNA. Triapine in combination with cisplatin, gemcitabine and other chemotherapeutic agents is currently being tested in clinical trials for solid tumors and leukemias (http://clinicaltrials.gov/ct2/results?term=triapine). Knockdown of RNR subunit expression by siRNAs has been attempted and although difficulties related to stability and target delivery complicate feasibility of use, there may be some promise with this approach. Clinical trials using GTI-2040, a 20-mer phosphorothioate oligodeoxynucleotide in combination have been completed in various solid cancers and myeloid leukemias (http://clinicaltrials.gov/ct2/results?term=GTI-2040).

There are known differences between NSCLCs from smokers and non-smokers, as well as men and women. However, high expression of RRM2 being more strongly associated with worse outcome in women and non-smokers is not easily explained. The mechanisms may be different in each group. Relevant to this, in colon cancer, Liu et al found a dramatic increase in the hazard ratio for RRM2 in a subgroup of patients who had a mismatch repair gene-deficiency [20]. Mutations resulting from imbalanced dNTP levels which evade proof-reading mechanisms can be repaired through the mismatch repair system [49]. Thus dysfunctional activity affecting both aspects of DNA replication may worsen patient survival outcomes.

In summary, immunohistochemical evaluation of RRM2 indicates that it has strong prognostic significance in some subsets of NSCLC patients (primarily women, non smokers and former smokers quitting longer than 10 years) but not others (men, current smokers and those who had stopped smoking for shorter periods of time). Originally we looked at the patient population as a whole, then separated out the subgroups where the outcome differences were particularly strong. Several publications previously reported that higher levels of RRM1 and/or RRM2 in NSCLC were associated with worse response to chemotherapeutic agents such as gemcitabine [28]. We did not have the information needed to examine RRMs and outcomes associated with chemotherapeutic agents in our study. From our results and the image quality (Fig 1) of the immunohistochemical staining, RRM2 may prove to be useful as a prognostic surgical pathology tool for NSCLC in certain patient groups, but further validation studies will be needed.

## Supporting Information

S1 Fig Kaplan-Meier curves for combined RRM1 and RRM2 expression. The differences in survival in women and non-smokers / long term former smokers were more consistently affected by RRM2 expression than RRM1. Kaplan-Meier curves for high and low expression of RRM1 and RRM2 both split at median levels: A) women; B) men; C) non-smokers + former smokers quitting ≥ 10 years; D) other smokers (current + former smokers quitting 10 years or less).
(EPS)

S1 Table RRM1 and RRM2 pooled data. (XLSX)

S2 Table RRM1 unpooled data. (XLSX)

S3 Table RRM2 unpooled data. (XLSX)
